# Immune monitoring of allograft status in kidney transplant recipients

**DOI:** 10.3389/fneph.2023.1293907

**Published:** 2023-11-08

**Authors:** Hwarang S. Han, Michelle L. Lubetzky

**Affiliations:** Division of Nephrology, Department of Internal Medicine, Dell Medical School, University of Texas at Austin, Austin, TX, United States

**Keywords:** kidney transplant, allograft rejection, immune monitoring, blood test biomarkers, urine biomarker, infection immunology, post transplant care, biopsy alternative

## Abstract

Kidney transplant patients require careful management of immunosuppression to avoid rejection while minimizing the risk of infection and malignancy for the best long-term outcome. The gold standard for monitoring allograft status and immunosuppression adequacy is a kidney biopsy, but this is invasive and costly. Conventional methods of allograft monitoring, such as serum creatinine level, are non-specific. Although they alert physicians to the need to evaluate graft dysfunction, by the time there is a clinical abnormality, allograft damage may have already occurred. The development of novel and non-invasive methods of evaluating allograft status are important to improving graft outcomes. This review summarizes the available conventional and novel methods for monitoring allograft status after kidney transplant. Novel and less invasive methods include gene expression, cell-free DNA, urinary biomarkers, and the use of artificial intelligence. The optimal method to manage patients after kidney transplant is still being investigated. The development of less invasive methods to assess allograft function has the potential to improve patient outcomes and allow for a more personalized approach to immunosuppression management.

## 1 Introduction

Kidney transplantation is the treatment of choice for patients with end-stage renal disease (ESRD) (1). However, kidney transplant recipients require lifelong immunosuppression to prevent rejection. Acute rejection during the first year after transplantation is a major risk factor for graft loss (2). Immunosuppression management has traditionally focused on preventing early rejection, requiring high dosages of medications that may be appropriate for some patients but can lead to unwanted side effects in others. Although this strategy has reduced the rates of early rejection, long-term outcomes have not improved substantially (3, 4). Calcineurin inhibitors, while key to reducing rejection rates, are also known to cause chronic allograft changes and higher doses are associated with increased risks of transplant associated infections (5). Improved methods are needed to individualize immunosuppression for each patient to ensure adequate coverage, while reducing the risks of chronic allograft changes and infection.

Typically, allograft dysfunction is signaled by a rise in serum creatinine or proteinuria. That said, by the time clinical signs arise, significant damage in the allograft may have already occurred. Protocol biopsy studies have routinely demonstrated the presence of rejection in otherwise clinically stable patients (6, 7). Immunosuppression adjustment has typically been reactionary, with doses being increased in the setting of rejection and lowered in the setting of infection or cancer. Increasing rates of post-transplant lymphoproliferative disorder (PTLD) and infections are seen as consequences of being over immunosuppressed (8, 9). Ultimately finding the right balance will require an individualized approach that will utilize many different tools available to transplant physicians. This review will discuss the current available methods for allograft monitoring and discuss novel methods that can be used together to improve post-transplant management.

## 2 Invasive methods of allograft monitoring

The gold standard for diagnosis of allograft dysfunction is the kidney biopsy. The procedure is invasive and not without risk of complications (10). A study of 2,514 kidney transplant biopsies over 5 years found that minor complications occurred in about 20% of cases, and major complications occurred in about 1.9% of cases (11). Minor complications included transient hematuria, small hematoma, and small fistula formation. Major complications included needing a blood transfusion, hospitalization, interventional radiology procedure, and surgical intervention. Major complications are more common in for-cause biopsies when compared to protocol biopsies (2.7% and 0.3%, respectively). Additionally, there is great variability when it comes to interpretation of biopsy results. Histological assessment has its limitations and pathology readings have been noted to be subjective and inconsistent (12–14). Despite the limitations and risks of biopsy, gene expression and histologic analysis of biopsy tissue can help clinicians better interpret and utilize information gained from tissue samples.

To improve histologic assessment and inter-reader variability, novel techniques have been developed that utilize gene expression profiles of kidney biopsy tissue. Gene expression of biopsy tissue can provide important information in terms of both diagnosis and prognosis of allograft dysfunction. The MMDx^®^ (molecular microscope diagnostic system, One Lambda, West Hills, CA) is a microarray-based test that uses machine learning to assess the risk of kidney transplant rejection. The test analyzes messenger RNA (mRNA) expression from a biopsy sample to identify patterns associated with rejection (15). The MMDx has been shown to have good correlation with histological findings, and it may be useful when histological results are borderline or inconclusive (16). MMDx has the potential to not only add additional information to the biopsy in question, but also lessen the need for repeat biopsies.

Analysis of histologic features has also been demonstrated to be a good predictor of allograft failure. A large multicenter study found that graft scarring, interstitial inflammation, tubulitis and micro-circulation inflammation in combination with the presence of donor specific antibodies (DSA) could predict rates of estimated glomerular filtration rate (eGFR) decline over time (17). The information gleaned from biopsies is likely to remain valuable, and when combined with newer noninvasive tests, it will provide even greater insights into the overall health of the allograft.

## 3 Non-invasive tests of allograft function

Graft damage and rejection can occur in the absence of an acute rise in serum creatinine. Historically, the only way to do determine changes in allograft status before graft dysfunction would be to perform protocol biopsies. Given the aforementioned risks of the procedure, less than half (46%) of the high-volume transplant centers (defined by annual kidney transplants greater than 50) in the United States perform protocol biopsies (18). This has led to the development of alternative tests that can non-invasively assess allograft function. Some are widely available for use in a clinical setting and others are less well known and/or not clinically available. These tests include measurements of gene transcripts in the blood, tests of lymphocyte function, donor derived cell free DNA analysis, alloantibodies, and monitoring for post-transplant infections (19).

### 3.1 Immune monitoring

Gene expression testing in the blood is a minimally invasive method that can be used to monitor kidney transplant patients. The most widely used is TruGraf® (Eurofins Transplant Genomics, Framingham, MA), which utilizes DNA microarray technology to determine whether a patient’s gene expression is more similar to a reference population with adequate immunosuppression than that with inadequate immunosuppression (20, 21). A study of 99 kidney transplant recipients with stable renal function and biopsy-confirmed rejection reported that gene expression (TruGraf) had a low positive predictive value (PPV) of 48% while the negative predictive value (NPV) was more acceptable at 89% for rejection (sensitivity was 71% and specificity was 75%) (22). AlloMap^®^ Kidney (CareDx, Brisbane, CA) uses next-generation sequencing and targeted RNA sequencing technology for gene expression profiling to assess immune quiescence (23). A study assessing 235 specimens (AlloMap Kidney) matched with histological results (66 rejection and 169 without rejection) from 222 patients showed NPV of 87% to 95%, but PPV was only 18% to 40% for allograft rejection (sensitivity was 70% and specificity was 66%) (24). Another gene expression test available for assessing rejection is the kSORT^®^ (kidney Solid Organ Response Test, Immucore, Norcross, GA). The kSORT looks at relative mRNA expression levels to detect patients who are at higher risk of rejection (25). Despite promising results in early studies, a large retrospective multicenter study of 1,763 samples from 1,134 patients found that kSORT could not be validated for acute rejection in the first year after transplantation (p = 0.46) (26). The kSORT is being refined to improve its performance (27). Although the above tests are promising, utilization s limited; given that they have a low PPV and high NPV, they are mostly helpful when the result is negative.

The use of urinary biomarkers for immune monitoring is an ideal choice because the kidney essentially acts like a “flow cytometer” filtering graft infiltrating cells into the urinary space (28). Some of the first studies of mRNA expression in the urine looked at expression of granzyme B and perforin, which are both integral parts of the cytotoxic T-cell response in allograft rejection. These studies found that elevated levels of granzyme B and perforin were potentially diagnostic of acute rejection (29, 30). Since this discovery, many other urinary markers are being studied with promising potential (31). A study of 83 kidney transplant recipients indicated that forkhead box P3 (FOXP3) mRNA in urine could be used to predict acute rejection with 90% sensitivity and 73% specificity (32). One of the largest urinary biomarkers studies to date evaluated 485 kidney transplant recipients and found that CD3ϵ mRNA, interferon-inducible protein 10 (IP-10) mRNA, and 18S ribosomal RNA (rRNA) levels in the urine could distinguish between T-cell mediated rejection (TCMR) and no rejection during the first-year post-transplant (33). Additionally, this study demonstrated that these urinary markers began to rise before any clinical signs of graft dysfunction and therefore could predict the development of acute rejection if monitored early in the post-transplant course. Urine mRNA profiling has also been shown to be able to identify both types of rejections, interstitial fibrosis, tubular atrophy, and BK nephropathy (34–36). Unfortunately, there are currently no commercially available urinary biomarker tests.

Tests of lymphocyte function have shown some promise in assessing the overall immunosuppression of a given patient. ImmuKnow^®^ (immune cell function assay, Eurofins Viracor, Lenexa, KS) measures the concentration of adenosine triphosphate (ATP) from CD4+ T-cells after stimulation to monitor the immune response of transplant patients (37). The test assigns patients into three categories based on their intracellular ATP levels: low (<225 ng/ml), moderate (226-524 ng/ml), and strong (>524 ng/ml). Lower ATP levels were correlated with “over immunosuppressed state” and increased risk of infection while the higher ATP levels were correlated with rejection, suggesting that patients should be aimed towards the moderate zone (38). However, there is significant overlap between stable and infected patients in the moderate range, which limits the test’s generalizability (39, 40). Although this test is clinically available, its usage has been limited. There are other functional cell-based tests. The Pleximark^®^ (Plexision, Pittsburgh, PA) looks at allo-antigen-specific T-cytotoxic memory cells but has only shown to measure likelihood of TCMR (41). A test looking at allo-antigen-specific B-cells (PlexABMR^®^, Plexision, Pittsburgh, PA) is being developed to be able to measure the risk of antibody-mediated rejections (ABMR).

One of the more promising technologies that is clinically available is the use of donor derived cell-free DNAs (dd-cfDNA) testing. cfDNA is non-encapsulated DNA that can be released after cells have been injured. In solid organ transplantation, dd-cfDNA has been investigated as a potential biomarker for allograft rejection (42). Single-nucleotide polymorphisms (SNPs) are used to differentiate dd-cfDNA from recipient-cfDNA. Three commercially available dd-cfDNA tests are in the United States: Allosure^®^ (CareDx, Brisbane, CA) which analyzes 405 SNPs across all somatic chromosomes, Prospera^®^ (Natera, Austin, TX) which analyzes over 13,000 SNPs of 4 select chromosomes, and TRAC^®^ (transplant rejection allograft check, Eurofins Viracor, Lenexa, KS) which uses a proprietary number of SNPs (43–45). Recently, the ADMIRAL study assessed 1,094 kidney transplant recipients and followed for 3 years. Results revealed that dd-cfDNA (Allosure) levels were significantly higher in patients with clinical and subclinical rejection than in patients without rejection. The mean dd-cfDNA level was 0.23% in the absence of rejection and 1.6% in the presence of rejection (p < 0.001) (46). PPV for rejection was only 59% and the NPV was better at 87% when threshold percentage of dd-cfDNA was 1% (sensitivity was 64% while specificity was 73%). These results suggest that dd-cfDNA is a promising biomarker for graft rejection, but it is worth noting its limitations. Evidence from the DART study showed that dd-cfDNA may not reliably identify TCMR type 1A or borderline rejections (47). Allograft tissue breakdown caused by other factors, such as BK nephropathy and urinary tract infections, can increase dd-cfDNA levels in the absence of rejection (48). The PPV is relatively low, meaning that a positive test result does not always indicate rejection. However, the high NPV suggests that a negative test result is a good indication that there is no rejection.

Test combining dd-cfDNA and gene expression have emerged as a solution to improve the diagnostic capabilities. The biggest benefit of these combined tests is the improved PPV. A post-hoc analysis study of 208 patients with biopsy and blood sample results showed that the PPV improved to 81% when both gene expression (TruGraf) and dd-cfDNA (TRAC) were positive (NPV of 88% when both tests were negative) (49). The study also suggested that gene expression profiling might detect TCMR better while dd-cfDNA might better detect ABMR. However, it is worth mentioning that there have been recent restrictions imposed on Medicare reimbursement for dual biomarkers, which will likely affect their availability (50).

### 3.2 Monitoring for alloantibodies

Donor-specific antibodies (DSAs) are produced by the recipient against the donor’s human leukocyte antigens (HLA) molecules. DSAs can cause ABMR, which is a major cause of graft loss after kidney transplantation (51). DSAs can be identified before kidney transplantation, and these pre-existing DSAs are associated with an increased risk of early rejection. DSAs that are found for the first time after kidney transplantation are called *de novo* DSAs (dnDSAs). The presence of DSA has been associated with declined eGFR over time (17). DSAs can cause allograft injury by activating complement cascade or by non-complement mediated mechanisms (52). Around 13% to 30% of kidney transplant recipients develop dnDSA, and presence of dnDSA is associated with poorer outcomes (53). For example, one study following 508 renal transplant patients (64 with dnDSA) reported that recipients without dnDSA had eGFR decline of 0.65 mL/min/1.73m2 per year and presence of dnDSA led to eGFR decline of 3.63 mL/min/1.73m2 per year (p < 0.001) (54). The risk factors for developing dnDSAs include inadequate immunosuppression but also infections such as BK virus and cytomegalovirus (55). Current guidelines from the Transplantation Society recommend monitoring for DSA in patients with DSA pre-transplant when immunosuppression is being reduced (in the setting of infection or for other reasons), when there is concern for non-adherence, and in patients with a rejection episode (56). Some evidence suggests that the presence of DSAs alone, without biopsy-proven rejection or acute inflammation, may not be associated with graft failure, highlighting our continued reliance on kidney biopsy (57). That said, the frequency of monitoring DSAs is at the discretion of the transplant center.

### 3.3 Monitoring for viral infections

BK polyomavirus is a common DNA virus that infects people of all ages and remains latent in the genitourinary cells (58). The abbreviation BK comes from the name of the first patient in whom the virus was isolated in 1971 (59). BK virus is normally benign in healthy individuals with functional T-cells but can cause tubulointerstitial nephritis, and in some cases, ureteral stenosis in transplant recipients (60, 61). Around 10% to 30% of kidney transplant recipients develop BK viremia within their first year of post-transplant (62). The treatment of choice for BK viremia is reduction in immunosuppression and therefore close monitoring for BK viremia in the early post-transplant period has now become standard of care.

Torque teno virus (TTV) is a non-pathogenic virus that is almost ubiquitous worldwide, with 90% of healthy individuals and up to 100% of transplant recipients infected (63). TTV is insensitive to conventional antiviral drugs used in transplantation, but it has the potential to be used as a marker of immune status in transplant recipients. TTV actively replicates and over 90% of the viruses are cleared by the immune system daily. T-cell function is thought to be crucial for viral control (63, 64). Observational studies have shown that low TTV load is associated with a higher risk of rejection, while high TTV load is associated with a risk of infection (65, 66). A multicenter prospective study is currently underway to assess the non-inferiority of TTV-guided immunosuppression in kidney transplant recipients (67).

## 4 Prognostication

Kidney transplant recipients could benefit from improved planning if graft loss can be predicted. As previously stated, histological assessment, gene expression profiling of biopsy, and the presence of DSA have shown potential for prognostication. Proteinuria has also commonly been used to predict poor prognosis (68). Other prediction tools may also be useful. A study recently examined four variables (age, sex, eGFR, and albuminuria) kidney failure risk equation (KFRE) in kidney transplant recipients (69). Study demonstrated that in patients with an eGFR of < 45 mL/min/1.73 m2, the KFRE predicted risk of ESRD in 2 and 5 years (concordance statistic of 0.88 and 0.83 respectively) at 1-year post-transplant.

### 4.1 Artificial intelligence

Artificial intelligence (AI) is a rapidly developing field with the potential to revolutionize medicine. One area where AI is being explored is in the prediction of kidney transplant outcome. The iBox (Integrative Box) is an AI-based scoring system, which was developed by analyzing 32 potential prognostic factors in 7,557 kidney transplant recipients (70). The score is computed by combining data from eGFR, proteinuria, histological scores, and DSA profiles to predict the risk of long-term kidney transplant loss (concordance statistic of 0.81). The iBox also has potential in its use as a surrogate endpoint for clinical trials to help advance novel therapies (71, 72). As AI technology continues to improve, it is likely that AI-based tools will become more widely used. More studies are needed to confirm the clinical utility of such tools and to investigate whether data from novel tests can improve these prognostic tools.

## 5 Conclusions

**Figure 1:**
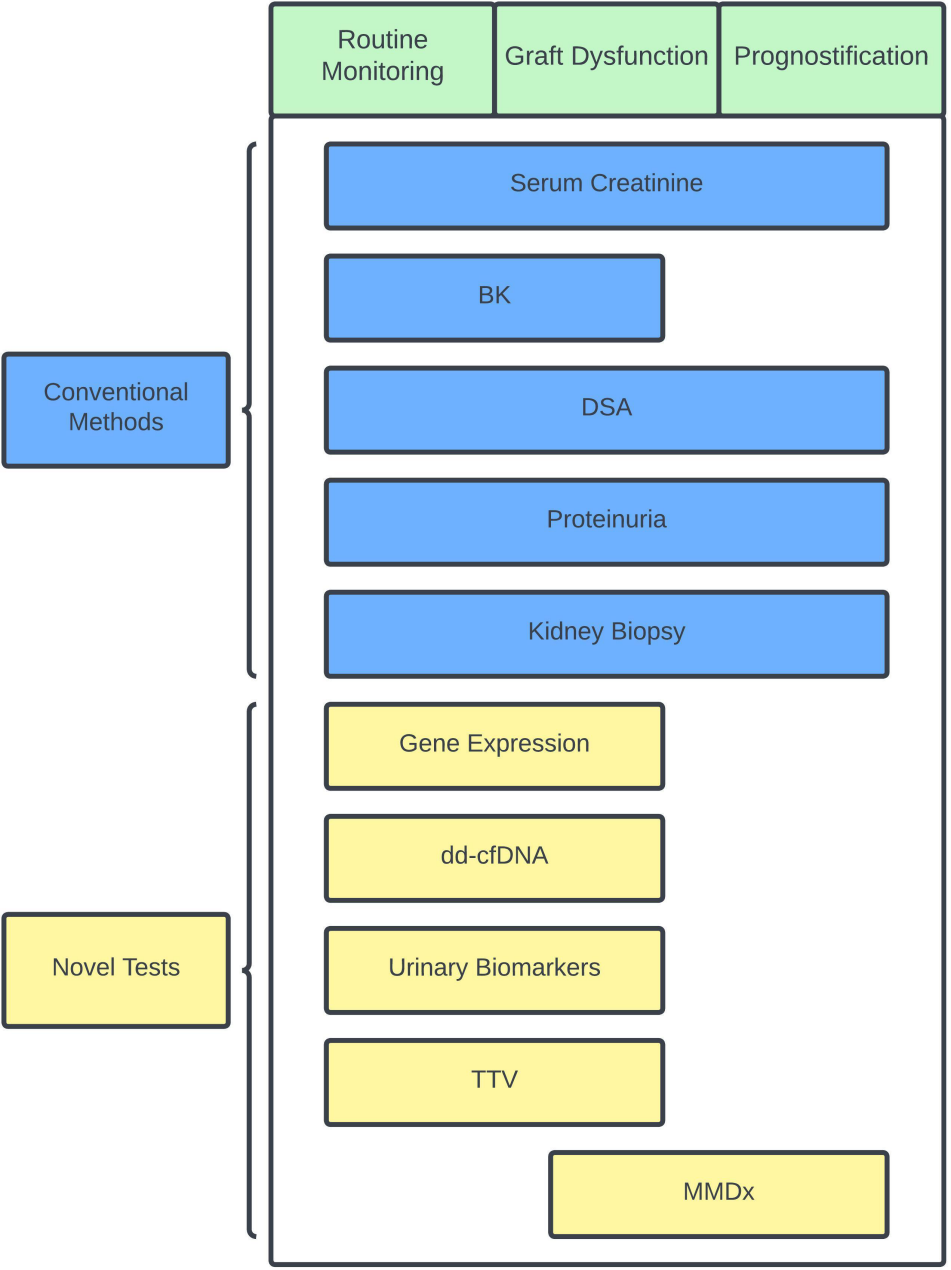
Schematic chart showing conventional and novel immune and status monitoring options during specific scenarios. Specific scenarios in green and conventional methods are shown in blue while novel tests are shown in yellow. BK, BK polyoma virus; dd-cfDNA, donor-derived cell-free DNA; DSA, donor-specific antibody; MMDx, molecular microscope diagnostic system; TTV, torque teno virus.

Improvements in immunosuppressive medications, antimicrobial regiments, and surgical techniques have improved short-term outcomes for kidney transplant recipients over the years. There is still a large gap in our knowledge about how to achieve better long-term outcomes. Personalizing immunosuppressive therapy may be one way to reduce late allograft losses, especially as the organ waitlist continues to grow. Currently, no single non-invasive test is accepted as a reliable alternative to histological assessment. The strong negative predictive values of dd-cfDNA and gene expression have potential to lessen the need for routine biopsies. Monitoring for the presence of DSA and viral infections can be also used as a surrogate marker of over- or under-immunosuppression. In combination with some of the currently available blood biomarker testing, clinicians can potentially identify clinically stable patients at low risk for rejection (normal dd-cfDNA/gene expression and no DSA), clinically stable patients at higher risk for rejection (dnDSA, abnormal dd-cfDNA/gene expression), and those who are over-immunosuppressed (BK viremia) so that adjustments to immunosuppression can be made accordingly and ideally before any graft damage occurs. **Figure 1** presents a schemata of post-transplant monitoring and how clinicians can potentially utilize the different tools available for allograft monitoring to help personalize immunosuppression. This review is not exhaustive and there are other methods to monitor immune status in kidney transplant recipients that are available and under investigation. Kidney transplant biopsy remains the gold standard for immune and allograft monitoring, and its overall assessment of allograft dysfunction will continue to improve as less invasive complementary methods emerge.

## Author contributions

HH: Writing – original draft, Writing – review & editing. ML: Supervision, Writing – review & editing.
